# The Role of Bovine Amniotic Membrane and Hydroxyapatite for the Ridge Preservation

**DOI:** 10.1155/2024/4053527

**Published:** 2024-09-14

**Authors:** Octarina Octarina, Elly Munadziroh, Fathilah Abdul Razak, Ekowati Handharyani, Meircurius Dwi Condro Surboyo

**Affiliations:** ^1^Department of Dental Material, Faculty of Dentistry, Universitas Trisakti, Jakarta 11440, Indonesia; ^2^Doctoral Program, Faculty of Dental Medicine, Universitas Airlangga, Surabaya 60132, Indonesia; ^3^Department of Dental Material, Faculty of Dental Medicine, Universitas Airlangga, Surabaya 60132, Indonesia; ^4^Department of Oral and Craniofacial Sciences, Faculty of Dentistry, Universiti Malaya, Kuala Lumpur 50603, Malaysia; ^5^Division of Pathology, School of Veterinary Medicine and Biomedical, Institute Pertanian Bogor University, Bogor 16680, Indonesia; ^6^Department of Oral Medicine, Faculty of Dental Medicine, Universitas Airlangga, Surabaya 60132, Indonesia

## Abstract

Ridge preservation is an important technique for maintaining the dimensions of the alveolar bone following tooth extraction, which is crucial for successful tooth rehabilitation. The combination of bovine amniotic membrane and hydroxyapatite has shown promise as a scaffold material containing growth factors that can stimulate osteogenic-related factors such as bone morphogenetic protein 2 (BMP2), Runt-related transcription factor 2 (RUNX2), and osteocalcin. This stimulation leads to collagen production and osteoblast proliferation, resulting in new bone formation. In this study, bovine amniotic membrane-hydroxyapatite (BAM-HA) composites were prepared using three different ratios of bovine amniotic membrane and hydroxyapatite (2 : 3, 3 : 7, 7 : 13). Thirty *Sprague–Dawley* rats had their first incisors extracted, and different types of BAM-HA were applied for ridge preservation. The control group received no treatment, while the positive control group was given xenograft. After 14 and 28 days, the animals were sacrificed, and immunohistochemical analysis was performed to evaluate the expression of BMP2, RUNX2, and osteocalcin. Additionally, a histological examination was conducted to analyse collagen thickness and osteoblast cell proliferation. The results demonstrated that the application of BAM-HA significantly increased collagen density, osteoblast cell proliferation, and the expression of BMP2, RUNX2, and osteoclacin compared to the control group (*p* < 0.05) on both days 14 and 28. Furthermore, increasing the hydroxyapatite content in the composite was found to enhance collagen thickness, osteoblast cell proliferation, and the expression of osteogenic-related factors. These preliminary findings suggest that the combination of BAM-HA can be used for ridge preservation to prevent further bone resorption following tooth extraction.

## 1. Introduction

Dental implant restoration is currently considered a viable option for patients who have experienced partial or complete tooth loss [1]. Dental implants' success depends on the alveolar bone's quantity and quality [2]. In successful dental implants, the quality of the alveolar bone can be considered good if the resorption process is less than 1 mm within a year [3]. Therefore, strategies for preserving the alveolar bone after dental extraction are necessary to achieve and maintain the quality of the alveolar bone. The ridge preservation represents a challenging technique that involves active biomaterials or autologous bone placed in the alveolar socket after the tooth extraction [4]. The goal of ridge preservation is to maintain the alveolar bone and minimise or prevent alveolar bone resorption [5].

The amniotic membrane is one potential osteoinductive biomaterial for bone healing [6]. One of the common amniotic membranes used is a bovine amniotic membrane (BAM), which contains various types of collagen and growth factors [7]. The bovine amniotic membrane shares similarities with the human amniotic membrane [8]. The growth factors found in the amniotic membrane include epidermal growth factor (EGF), transforming growth factor alpha and beta (TGF-*α* and TGF-*β*), keratinocyte growth factor (KGF), hepatocyte growth factor (HGF) and basic fibroblast growth factor (bFGF) [9]. In addition, the extracellular matrix of the bovine amniotic membrane consists of various types of collagen, laminin, nidogen, fibronectin, and proteoglycans [10]. Several studies have shown that the amniotic membrane expresses C–X–C chemokine receptor type 4 (CXCR-4), monocyte chemoattractant protein-1 (MCP-1), osteocalcin, and cathepsin K (CatK), indicating its osteoinductive ability [6].

Hydroxyapatite (HA) is a biomaterial that contains stable calcium phosphate salts with a chemical formula of Ca_10_ (PO_4_)_6_ (OH)_2_, plays a role in bone formation, or substitution and can be used for ridge preservation [11]. The role of hydroxyapatite in bone is to act as a scaffold [12], provide osteoconductive properties, and stimulate osteoblast differentiation during bone remodelling [13]. Combining the bovine amniotic membrane with hydroxyapatite (BAM-HA) is interesting because these materials may possess synergistic abilities and proceed with bone regeneration. The first research showed that the combination of bovine amnion membrane and hydroxyapatite with a ratio of 7 : 13 produced the desired characteristics, such as a pore size of 155.625 *μ*m, porosity of 89.23% [14], and maximum swelling ability [15]. This characteristic depends on the ratio of the amniotic membrane; an increased ratio leads to an increase in pore size and porosity [14].

The synergistic combination of bovine amniotic membrane and hydroxyapatite holds great promise as a material for ridge preservation, effectively maintaining the quality and quantity of alveolar bone. The study employed histological and immunohistochemical analysis to examine the proliferation phase [16] and remodelling phase [5]. In vivo, tests were conducted to evaluate collagen thickness, osteoblast cell proliferation and the expression of BMP2, RUNX2, and osteocalcin. Collagen, an integral component of wound healing, plays a crucial role in the early stages of bone healing [17]. Osteoblast cells serve as markers for alveolar bone healing and express bone-forming proteins. BMP2, known for its ability to induce bone formation [18], and RUNX2, which promotes osteoblast differentiation and stimulates osteocalcin production, were also analyzed. Osteocalcin, in turn, facilitates calcium binding to the bone matrix, aiding in late-stage osteoblast differentiation [19]. To investigate the potential of bovine amniotic membrane and hydroxyapatite in various ratios (2 : 3, 3 : 7, and 7 : 13) and find the optimum combination for alveolar ridge preservation, the current study focused on analyzing collagen density, osteoblast activity, and osteogenic markers, including BMP2, RUNX2, and osteocalcin.

## 2. Materials and Methods

### 2.1. Bovine Membrane Amnion Preparation

The bovine amniotic membrane was obtained from a female *Bos javanicus domesticus*. The bovine amniotic membrane was initially washed to remove blood clots using a 0.05% saline solution, with each washing step lasting 10 minutes. Subsequently, it was further washed with Aquadest until the saline solution became clear. The cleaned bovine amniotic membrane was then kept at a temperature of −80°C for 24 hours in a freezer. After that, freeze-drying was performed for 24 hours at −100°C. The resulting product was obtained in sheet form.

### 2.2. Hydroxyapatite Preparation

Cancellous bone samples (cancellous bone originating from the spongy part of the hump) were obtained from a seven-year-old female *Bos javanicus domesticus*. The bone samples were cut into small pieces and thoroughly washed with water. Subsequently, the bone pieces were placed in an ultrasonic shaker at 60°C to remove the fat content. After this process, the bones were washed again using Aquadest.

Following the washing step, the bone pieces were air-dried and then subjected to a furnace at 1000°C for one hour for burning. After burning, the bones were washed 3–4 times using Aquadest.

The bones were then dried again in an oven at a temperature range of 60–100°C until completely dry. Once thoroughly dried, the bones were ground into particles using a bone miller until they reached a particle size of 150 *μ*m.

### 2.3. Combination of Bovine Membrane Amnion and Hydroxyapatite

**Figure 1:**
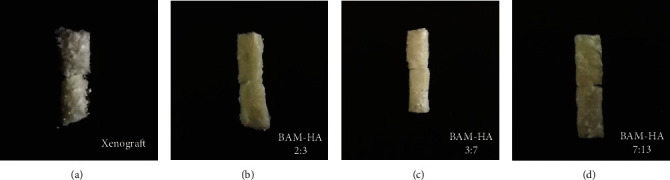
The combination of bovine amniotic membrane and hydroxyapatite in 3 different ratios 3 : 7 (b), 7 : 13 (c), 2 : 3 (d), and xenograft was used as a control (a).

The bovine amniotic membrane and hydroxyapatite (BAM-HA) combination was prepared with three different weight ratios of bovine amniotic membrane to hydroxyapatite, namely 2 : 3, 3 : 7, and 7 : 13 (weight/weight). A predetermined weight of amniotic membrane was soaked in 40 ml of 0.9% natrium chloride solution for five minutes. Subsequently, the bovine amniotic membrane was homogenised using a blender for 10 minutes until a homogenous amniotic slurry was obtained. The bovine amniotic slurry was then mixed with the previously prepared HA powder. The mixture was stirred until homogenous and transferred into a Petri dish with a diameter of 10 cm. The Petri dish was then stored in a freezer at −80°C for 24 hours, followed by freeze drying for another 24 hours at a temperature of −100°C. The resulting combination formed sponge-like structures, which were further sterilised with a gamma radiation dose of 25 Gy (Figure 1).

### 2.4. Animals

A total of 30 male *Sprague–Dawley* rats (four months old, 300 g) were subjected to a seven-day adaptation period before the commencement of the experimental treatments. Each rat was individually housed in covered cages (dimensions: 39 × 42 × 15 cm). The rats had sufficient food, water, ventilation, and appropriate lighting conditions. They were fed twice daily, with a total of 20 grammes of standard diet per rat. Additionally, the rats received deworming, antiectoparasites, and vitamins as additional supplements to support their overall health.

### 2.5. Alveolar Bone Regeneration Model

The alveolar bone regeneration model was established in rats, beginning with the extraction of the first mandibular incisor on the right side. During the extraction process, the rats were anaesthetised by intraperitoneal administration of ketamine and xylazine (2 : 1, v/v).

The extraction procedure involved initially destroying the periodontal ligament using a probe. Once the buccal, lingual, mesial, and distal aspects of the periodontal ligament were detached, the tooth was gently removed using a dental excavator until it was completely and instantly removed. After the completion of extraction, each rat was assigned to its respective treatment, as mentioned in Table 1.

Following the application of amnion membrane and hydroxyapatite in the socket, suturing was performed using three stitches of catgut (0.4 *µ*) thread. The rats were administered antibiotics (ampicillin) and analgesics (paracetamol) to mitigate potential side effects from the extraction, such as swelling and pain.

### 2.6. Alveolar Bone Regeneration Analysis

After 14- and 28 days post-tooth extraction, all animals were euthanised using an overdose of ketamine (95 mg/kg body weight) and xylazine (5 mg/kg body weight). The mandible was collected, and sagittal dissection was performed on the alveolar bone in the anterior tooth region. The alveolar bone was then immersed in 10% neutral-buffered formalin for fixation, followed by decalcification using 30% ethylenediaminetetraacetic acid (EDTA) for seven days. The tissue samples were subsequently embedded in paraffin and sectioned into 5 *µ*m thick transverse slices for collagen density, osteoblast, BMP2 expression, RUNX2 expression, and osteocalcin expression.

#### 2.6.1. Osteoblast Analysis

Osteoblasts were analyzed in the alveolar bone using hematoxylin and eosin tissue staining. The quantification of osteoblasts was performed by counting the number of osteoblasts at the edge of the alveolar bone socket under a light microscope at a magnification of 400 x. Five different regions of interest were examined in each preparation or sample to ensure a representative analysis.

#### 2.6.2. Collagen Density Analysis

The collagen density analysis was performed by evaluating the thickness of collagen fibers in histologically stained tissue exhibiting a bluish hue following Masson's Trichrome staining. The quantification of collagen density was conducted based on a scoring system according to the following criteria: 0 (absence of collagen fiber appearance), 1 (very thin/few collagen fibers observed), 2 (thin and scattered collagen fibers observed) and 3 (thick and widely distributed collagen fibers observed). A light microscope at a magnification of 400 x and five different regions of interest were examined in each preparation or sample to ensure representative analysis.

#### 2.6.3. BMP2, RUNX2, and Osteocalcin Expression

BMP2, RUNX2, and osteocalcin expression were analyzed in alveolar bone using indirect immunohistochemistry staining. The primary antibodies were BMP2 (1 : 100), RUNX2 (1 : 50), and osteocalcin (1 : 200) from Affinity Biosciences, Inc., USA. The 3,3′-diaminobenzidine (DAB) system was used as the secondary antibody *(Universal HRP Excell Stain, Biogear, Life Science)*. Haematoxylin 560 was used as the counterstain *(Leica Biosystem).*

The quantification of BMP2 and osteocalcin was performed by counting the chondrocytes showing immunoreactivity, visualized as brown staining in the nucleus and cytoplasm. Similarly, the quantification of RUNX2 was carried out by counting the osteocytes showing immunoreactivity, visualized as brown staining in the nucleus and cytoplasm. This analysis was performed in five different regions of interest using a ZEISS AXIO SCOPE AI microscope equipped with an AxioCam digital camera. The images were captured and analyzed using Zen 3.4 software at a magnification of 40 x.

### 2.7. Statistical Analysis

The statistical analysis was conducted using a one-way ANOVA to analyse the differences in osteoblasts, collagen, BMP2, RUNX2, and osteocalcin expression after administration of different ridge preservation (control, xenograft, BAM-HA 2 : 3, BAM-HA 3 : 7 and BAM-HA7 : 13). A significance level of *p* < 0.05 was used to determine statistically significant results. SPSS version 24 (IBM SPSS Statistic 24 for Windows, New York, NY, USA) was used for the analysis.

## 3. Results

### 3.1. Collagen Number

**Figure 2:**
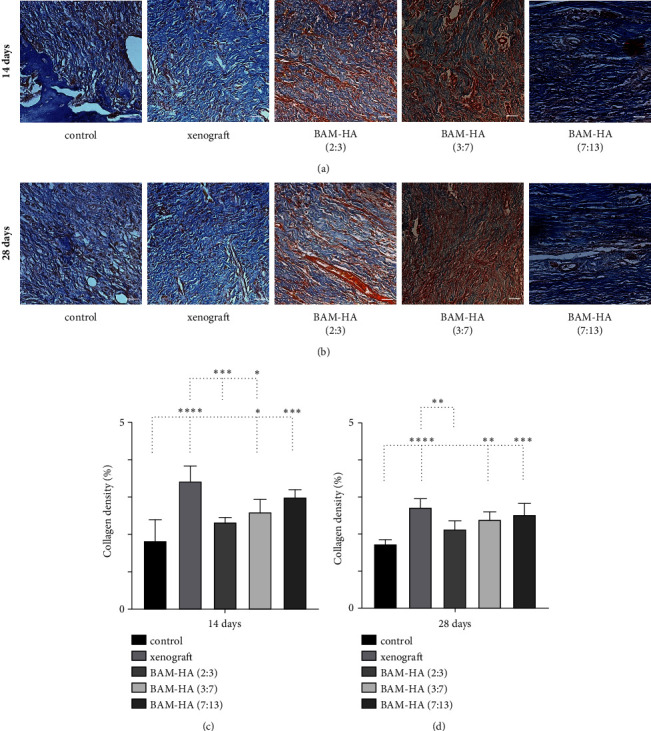
The collagen analysis in alveolar bone using histopathology analysis using Masson Trichome staining. (a and b) The visualization of alveolar bone in each group in 400 x magnification. The collagens were stained blue in colour. (c and d) The quantification of collagens density in 14 days and 28 days of observation using Image*J* software. Asterix in C and D mean different collagen density among groups analyzed using one-way ANOVA test and post hoc test. ^∗^*p* < 0.05; ^∗∗^*p* < 0.01; ^∗∗∗^*p* < 0.001; ^∗∗∗∗^*p* < 0.0001; ns = not significant (not shown). Scale bar 100 *μ*m.

The collagen density showed a higher number in amnion membrane hydroxyapatite (3 : 7) and (7 : 13) compared to the control (*p* < 0.05 and *p* < 0.001) on days 14 and 28 of observation. Compared to xenograft density was lower in amnion membrane hydroxyapatite (2 : 3) and (3 : 7) (*p* < 0.05 and *p* < 0.001) (Figure 2).

### 3.2. Osteoblast Number

**Figure 3:**
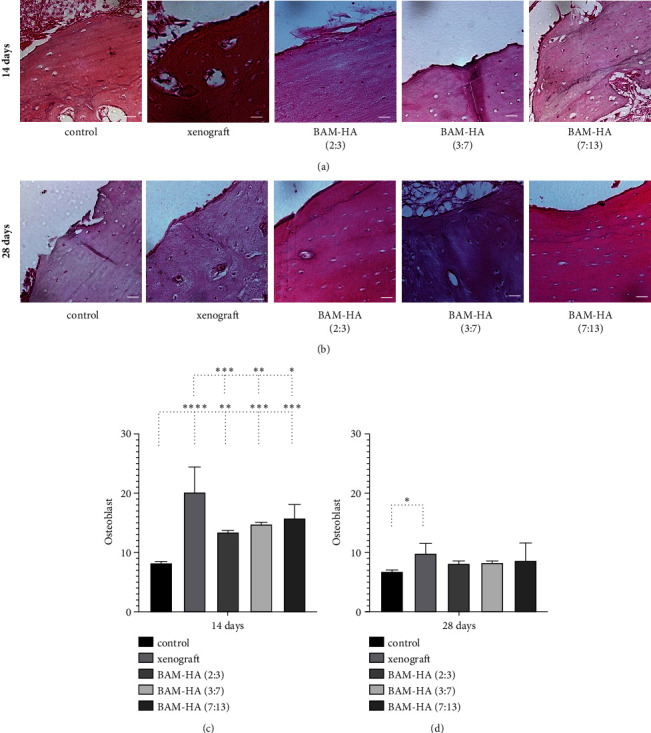
The osteoblast analysis in alveolar bone using histopathology analysis using hematoxyline-eosin staining. (a and b) The visualization of alveolar bone in each group in 400 x magnification. (c and d) The quantification of osteoblast in 14 days and 28 days of observation. Asterix in C and D mean different osteoblast among groups analyzed using one-way ANOVA test and post hoc test. ^∗^*p* < 0.05; ^∗∗^*p* < 0.01; ^∗∗∗^*p* < 0.001; ^∗∗∗∗^*p* < 0.0001; ns = not significant (not shown). Scale bar 100 *μ*m.

The osteoblast showed a higher number in all combinations of amnion membrane and hydroxyapatite compared to the control (*p* < 0.0001) on day 14 of observation. Compared to xenograft, the osteoblast showed lower levels in all groups of amnion membrane hydroxyapatite (*p* < 0.05, *p* < 0.001, *p* < 0.0001). In the 28 days of observation, there was no significant difference between control xenograft and all amnion membrane hydroxyapatite groups (Figure 3).

### 3.3. The BMP2 Expression

**Figure 4:**
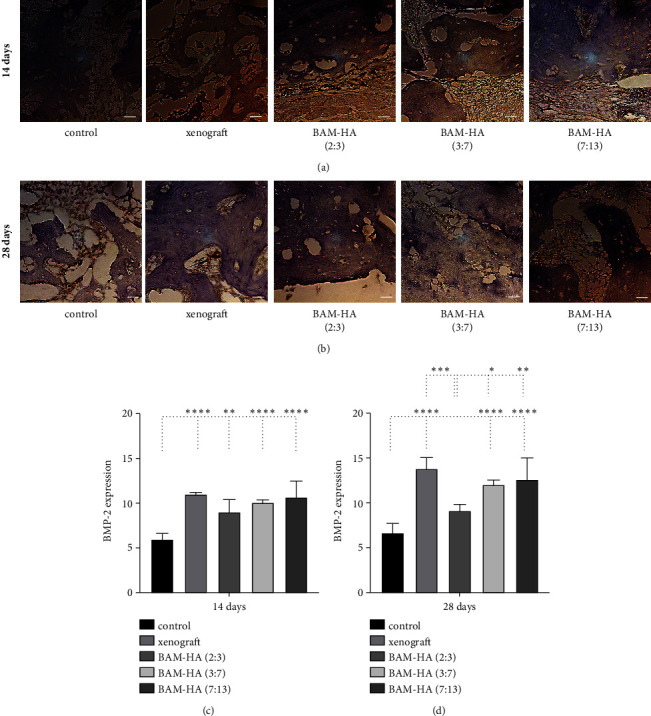
The BMP2 expression in alveolar bone using indirect immunohistochemistry staining. (a and b) The visualization of alveolar bone in each group in 400 x magnification. (c and d) The quantification of BMP2 expression in 14 days and 28 days of observation. Asterix in C and D mean different BMP2 expression among groups analyzed using one-way ANOVA test and post hoc test. ^∗^*p* < 0.05; ^∗∗^*p* < 0.01; ^∗∗∗^*p* < 0.001; ^∗∗∗∗^*p* < 0.0001; ns = not significant (not shown). Scale bar 100 *μ*m.

The BMP2 expression showed a higher number in combination with amnion membrane hydroxyapatite (3 : 7) and (7 : 13) compared to the control (*p* < 0.01 and *p* < 0.0001) on days 14 and 28 of observation. Compared to xenograft, BMP2 expression showed no significant difference in all groups of amnion membrane hydroxyapatite. In 28 days of observation, a combination of amnion membrane hydroxyapatite (7 : 13) showed a higher expression compared to a combination of amnion membrane hydroxyapatite (3 : 7) (Figure 4).

### 3.4. The RUNX2 Expression

**Figure 5:**
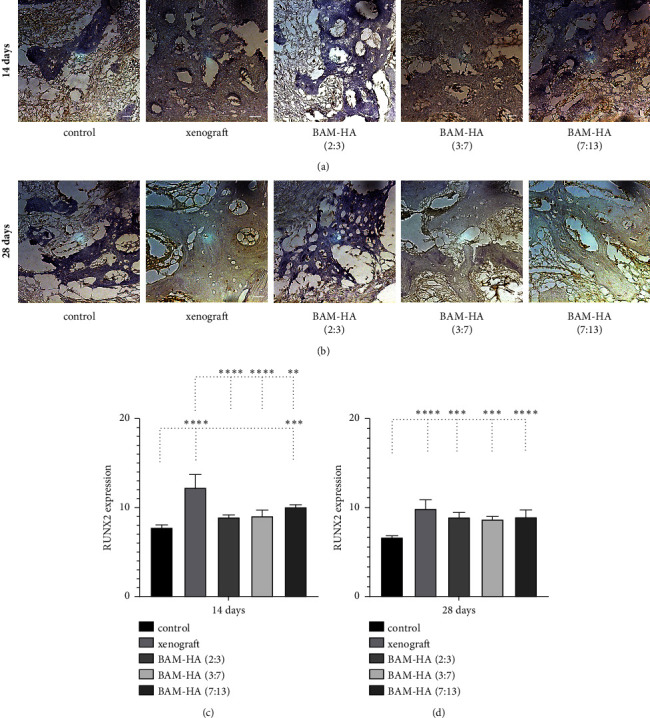
The RUNX2 expression in alveolar bone using indirect immunohistochemistry staining. (a and b) The visualization of alveolar bone in each group in 400 x magnification. (c and d) The quantification of RUNX2 expression in 14 days and 28 days of observation. Asterix in C and D mean different RUNX2 expression among groups analyzed using one-way ANOVA test and post hoc test. ^∗^*p* < 0.05; ^∗∗^*p* < 0.01; ^∗∗∗^*p* < 0.001; ^∗∗∗∗^*p* < 0.0001; ns = not significant (not shown). Scale bar 100 *μ*m.

The RUNX2 expression showed a higher number in combination with amnion membrane hydroxyapatite (7 : 13) compared to the control (*p* < 0.001) on day 14 of observation. Compared to xenograft all combinations with amnion membrane hydroxyapatite showed lower RUNX2 expression (*p* < 0.01 and *p* < 0.0001). In 28 days, the RUNX2 expression showed a higher number in all combinations of amnion membrane hydroxyapatite compared to the control (*p* < 0.001), but was not different when compared with the xenograft groups (Figure 5).

### 3.5. The Osteocalcin Expression

**Figure 6:**
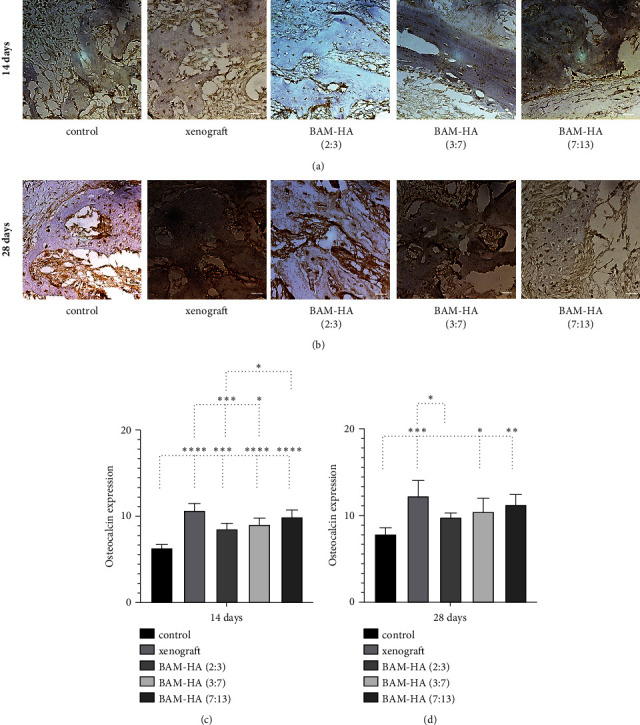
The osteocalcin expression in alveolar bone using indirect immunohistochemistry staining. (a and b) The visualization of alveolar bone in each group in 400 x magnification. (c and d) The quantification of osteocalcin expression in 14 days and 28 days of observation. Asterix in C and D mean different osteoclacin expression among groups analyzed using one-way ANOVA test and post hoc test. ^∗^*p* < 0.05; ^∗∗^*p* < 0.01; ^∗∗∗^*p* < 0.001; ^∗∗∗∗^*p* < 0.0001; ns = not significant (not shown). Scale bar 100 *μ*m.

The osteocalcin expression showed a higher number in all combinations with amnion membrane hydroxyapatite compared to the control (*p* < 0.001) on day 14 of observation. But, when compared with xenograft, the osteocalcin expression was lower in all groups of amnion membrane hydroxyapatite (*p* < 0.05 and *p* < 0.001). In 28 days of observation, only a combination of amnion membrane hydroxyapatite (3 : 7) and (7 : 13) had a higher osteocalcin expression compared to the control (*p* < 0.05 and *p* < 0.01) (Figure 6).

## 4. Discussion

**Figure 7:**
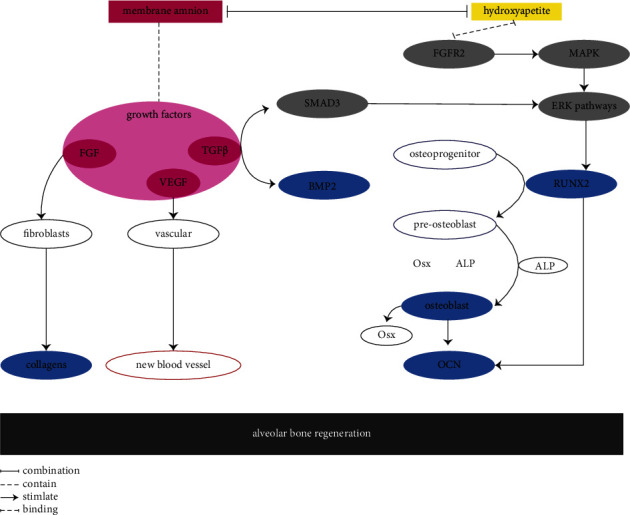
The possible mechanism combination amnion membrane–hydroxyapatite in alveolar bone regeneration.

Collagen plays a crucial role in the bone regeneration process. Collagen is the main protein component of the extracellular matrix, which repairs damage and restores the structure and anatomical function of tissues [20]. The administration of BAM-HA results in higher collagen thickness compared to the control group. The significant increase in collagen thickness in the BAM-HA group is attributed to the presence of various growth factors and proteomic secretory leukocyte protease inhibitors (SLPI) [21]. SLPI also plays a role in stimulating growth factors such as epidermal growth factor (EGF), vascular endothelial growth factor (VEGF), fibroblast growth factor (FGF), transforming growth factor-*β* (TGF-*β*) and platelet-derived growth factor (PDGF) [22]. These growth factors, particularly EGF, regulate epithelial cell motility, affecting the rate of re-epithelialization and assisting wound contraction by stimulating fibroblast proliferation and migration to restore tissue integrity [23]. Additionally, TGF-*β* stimulates fibroblast activity in the secretion of fibroblast growth factor (FGF), which binds to tyrosine kinase receptors, leading to receptor autophosphorylation and subsequent phosphorylation of serine, threonine and tyrosine residues on specific target proteins such as Raf-1, MAPK/Erk kinase (MEK), and extracellular signal-regulated kinase-1 (ERK) [24]. Both FGF and TGF-*β* increase fibroblast proliferation, thereby enhancing collagen synthesis [25, 26]. Moreover, BAM contains collagen types I, III, IV, V, VI, and XV [27], which further contribute to collagen formation at the site of the wound [28] (Figure 7).

The increase in collagen thickness within the socket stimulates the activity of osteoblasts, which are pivotal in the process of bone regeneration. Osteoblasts serve as markers of alveolar bone healing by expressing bone-forming proteins [29]. Similarly, the application of BAM-HA demonstrates a higher number of osteoblasts compared to the control group. The increased number of osteoblasts may be a result of enhanced fibroblast proliferation and collagen synthesis within the bone socket. As the increase in collagen serves as a new tissue matrix, it is always accompanied by angiogenesis, the formation of new blood vessels [30]. The formation of new blood vessels indicates the entry into the proliferation phase, allowing mesenchymal stem cells to differentiate into osteoblasts to generate bone [31]. During the proliferation phase, preosteoblast progenitor cells exhibit alkaline phosphatase (ALP) activity and are considered preosteoblasts. The transition from preosteoblasts to osteoblasts is characterised by an increase in the expression of Osterix (Osx) and the secretion of bone matrix proteins. Additionally, osteoblasts undergo morphological changes, becoming larger and assuming a cuboidal shape [32].

The promotion of osteoblast activity is further facilitated by the presence of hydroxyapatite in the bovine amniotic membrane, where HA crystals serve as ligands that activate signalling receptors and enhance the expression of osteogenic transcription factors. Osteoblast stimulation by hydroxyapatite can occur through the extracellular signal-regulated kinase (ERK) signalling pathway [33]. Additionally, fibronectin present in the amnionic membrane can interact with hydroxyapatite and activate the ERK pathway [34]. The ERK signalling pathway can also be stimulated by the interaction of HA with the fibroblast growth factor receptor (FGFr). FGFr binds to ligands that play a role in activating mitogen-activated protein kinases (MAPKs) [35]. The increase in the number of osteoblasts observed through the BAM-HA interaction via the ERK signalling pathway indicates an accelerated stimulus for alveolar bone healing (Figure 7).

On the other hand, various osteogenic markers also exhibit increased expression in the group treated with BAM-HA. One of the observed osteogenic markers is BMP2. BMP2 is a protein known to induce bone formation and plays a role in various biological processes of osteoblasts, including proliferation, differentiation, and apoptosis [36]. The increased expression of BMP2 can be attributed to the presence of growth factors such as TGF-*β* in the bovine amniotic membrane, which can induce BMP2 expression. The BMP or TGF-*β* pathway is essential for osteogenesis both in vitro and in vivo [37]. When BMP2 binds to BMP receptors located in lipid rafts, caveolae, and clathrin-coated pits (CCPs), it activates the phosphorylation of BMPRII and BMPRIa. This leads to the activation of Smad signalling or non-Smad pathways. Non-Smad signalling pathways, including ERK, phosphatidylinositol 3-kinase (PI3K), and transforming growth factor-*β*-activated kinase 1/binding protein 1 (TAB1/TAK1), are activated. All these pathways contribute to the differentiation of mesenchymal stem cells (MSCs) and osteoprogenitors into osteoblasts [30, 38].

Another osteogenic marker is RUNX2, which is an important factor in osteogenesis and osteoblast differentiation [39]. The increased expression of RUNX2 in the BAM-HA group is also influenced by the initial role of TGF-*β* present in the bovine amniotic membrane, leading to an enhancement of osteoprogenitors [40]. TGF-*β* activates SMAD3 and induces RUNX2 expression through the ERK pathway, which transforms osteoprogenitors into preosteoblasts. Additionally, the presence of FGF in the bovine amniotic membrane can increase RUNX2 expression through FGFR1 receptors [41]. Through the upregulation of RUNX2 expression, there is also an increase in osteocalcin production by osteoblasts.

Osteocalcin, the third osteogenic marker observed in this study, is a non-collagenous protein initially identified in bone and dentin as a calcium-binding protein secreted by mature osteoblasts, namely, osteocytes [42]. The increase in osteocalcin in alveolar bone is a result of increased RUNX2, which acts as a master regulator of osteogenesis, controlling the expression of non-collagenous proteins such as osteocalcin that play a role in the final stages of osteoblast differentiation, matrix binding of calcium, and induction of mineralization [43]. The increased osteocalcin production is expected to promote mineralization and facilitate the process of alveolar bone regeneration.

Based on this research, the application of this biocomposite material is not limited to just being a ridge preservation material after extraction. However, it is also hoped that it will be able to act as ridge preservation to stimulate new bone and improve alveolar bone healing in cases of large bone defect, such as cases of mini crestal sinus removal, implant placement, and major craniofacial surgery [30, 38]. The limitation of this study is no radiographic examination to confirm the presence of new bone. This examination is needed for the future application for human application. In the other hand, alveolar bone healing takes a longer time, it can take months to years, so it requires observation of the research object for a longer time.

## 5. Conclusions

The application of BAM-HA in the socket after tooth extraction showed an increase in bone remodelling. A higher ratio of hydroxyapatite increases collagen density, osteoblast, and osteogenic-related factors such as RUNX2, BMP2, and osteocalcin. This initial finding suggests that a combination of amnion membrane and hydroxyapatite can be used as ridge preservation to maintain the quality and quantity of alveolar bone. In large bone defects, it is also hoped that this material can also stimulate bone healing because the clot is the most powerful bone growth factor and is autologous in that it provides all the cells for bone transformation.

## Figures and Tables

**Table 1:** The animal designated group treatment after tooth extraction.

Name of groups	Number of rats	Treatment^1^
Control	6	—
Xenograft^2^	6	Applied bio-oss collagen
A	6	Applied combination of amnion membrane and hydroxyapatite ratio 2 : 3
B	6	Applied combination of amnion membrane and hydroxyapatite ratio 3 : 7
C	6	Applied combination of amnion membrane and hydroxyapatite ratio 7 : 13

^1^The material applied has dimensions 3 × 10 mm. ^2^Geistlich bio-oss® collagen, Geistlich pharma AG, bahnhofstrasse, Wolhusen, Switzerland.

## Data Availability

The data are available on personal request to first author and corresponding author.

## Abbreviations

DAB: 3,3′-diaminobenzidine
ALP: Alkaline phosphatase
bFGF: Basic fibroblast growth factor
BMP2: Bone morphogenetic protein 2
BAM-HA: Bovine amniotic membrane-hydroxyapatite
CatK: Cathepsin K
CCPs: Clathrin-coated pits
CXCR-4: C–X–C chemokine receptor type 4
EDTA: Ethylenediaminetetraacetic acid
EGF: Epidermal growth factor
ERK: Extracellular signal-regulated kinase-1
FGF: Fibroblast growth factor
HGF: Hepatocytes growth factor
KGF: Keratinocyte growth factor
MEK: MAPK/Erk kinase
MSCs: Mesenchymal stem cells
MAPKs: Mitogen-activated protein kinases
MCP-1: Monocyte chemoattractant protein 1
Osx: Osterix
PI3K: Phosphatidylinositol 3-kinase
PDGF: Platelet-derived growth factor
RUNX2: Runt-related transcription factor 2
SLPI: Secretory leukocyte protease inhibitors
TGF-*α*: Transforming growth factor alpha
TGF-*β*: Transforming growth factor beta
Table1/TAK1: Transforming growth factor-*β*-activated kinase 1/binding protein 1
VEGF: Vascular endothelial growth factor.

## Additional Points

*Institutional Review Board Statement*. The research was conducted in accordance with all the provisions of the Institute for Research and Community Service IPB. The approval code for this research is 232–2022 IPB.
